# Admission Blood Glucose Is Associated With the 30-Days Mortality in Septic Patients: A Retrospective Cohort Study

**DOI:** 10.3389/fmed.2021.757061

**Published:** 2021-10-28

**Authors:** Xiaoyuan Wei, Yu Min, Jiangchuan Yu, Qianli Wang, Han Wang, Shuang Li, Li Su

**Affiliations:** ^1^Department of Cardiology, The Second Affiliated Hospital, Chongqing Medical University, Chongqing, China; ^2^Department of Breast and Thyroid Surgery, The Second Affiliated Hospital, Chongqing Medical University, Chongqing, China

**Keywords:** sepsis, diabetes, blood glucose, risk factor, MIMIC III

## Abstract

**Background:** Sepsis, as one of the severe diseases, is frequently observed in critically ill patients, especially concurrent with diabetes. Whether admission blood glucose is associated with the prognosis, and outcome of septic patients is still debatable.

**Methods:** We retrospectively reviewed and analyzed the demographic characteristics of septic patients in the Medical Information Mart for Intensive Care III (MIMIC III, version 1.4) between June 2001 and October 2012. The Chi-square and Fisher's exact tests were used for the comparison of qualitative variables among septic patients with different glucose levels and the 30-day mortality in septic patients with diabetes or not. Univariate and stepwise multivariate Cox regression analyses were used to determine the risk factors for 30-day mortality. Kaplan-Meier analysis was conducted to reveal the different 30-day survival probabilities in each subgroup.

**Results:** A total of 2,948 septic patients (910 cases with diabetes, 2,038 cases without diabetes) were ultimately included in the study. The 30-day mortality was 32.4% (956/2,948 cases) in the overall population without any difference among diabetic and non-diabetic septic patients (*p* = 1.000). Admission blood glucose levels <70 mg/dl were only observed to be significantly associated with the 30-day mortality of septic patients without diabetes (hazard ratio (HR) = 2.48, *p* < 0.001). After adjusting for confounders, age >65 years (HR = 1.53, *p* = 0.001), the Sequential Organ Failure Assessment (SOFA) score >5 (HR = 2.26, *p* < 0.001), lactic acid >2 mmol/L (Lac, HR = 1.35, *p* = 0.024), and platelet abnormality (<100 k/ul: HR = 1.49; >300 k/ul: HR = 1.36, *p* < 0.001) were the independent risk factors for 30-day mortality in septic patients with diabetes. In non-diabetes population, age >65 years (HR = 1.53, *p* < 0.001), non-White or non-Black patients (HR = 1.30, *p* = 0.004), SOFA score >5 (HR = 1.56, *p* < 0.001), blood glucose <70 mg/dl (HR = 1.91, *p* = 0.003), anion gap (AG) >2 mmol/L (HR = 1.60, *p* < 0.001), Lac (HR = 1.61, *p* < 0.001), urea nitrogen >21 mg/dl (HR = 1.45, *p* = 0.001), alanine aminotransferase (ALT, HR = 1.31, *p* = 0.009), total bilirubin >1.2 mg/dl (HR = 1.20, *p* = 0.033), and low hemoglobin (HR = 1.34, *p* = 0.001) were the independent risk factors for 30-day mortality.

**Conclusions:** Our results indicate admission blood glucose, especially in terms of <70 mg/dl, is the key signaling in predicting the worse 30-day survival probability of septic patients without diabetes, which could help clinicians to make a more suitable and precise treatment modality in dealing with septic patients.

## Introduction

As a major global public health problem, sepsis has been one of the leading causes of death among patients admitted to the critical care unit (ICU) (1–4). Reviewing the clinical data from 409 hospitals in the US, sepsis was present in nearly 6% (173,690/2,901,019 cases) of adult hospitalizations (5). It is a life-threatening condition that arises when the immune response of the body to the infection injures its tissues. According to the latest systematic review reports (170 studies included), the average 30-day sepsis mortality was 24.4%, and 90-day sepsis mortality was 32.2% (6) in developed countries. Moreover, the mortality rate and estimated economic burden of sepsis could be even higher in developing countries and low-income regions (3, 7). Compared with other non-inflammation diseases, septic patients undergo more complex pathophysiological changes that are associated with systemic inflammatory response and subsequent acute organ dysfunction (4).

Unlike genetic markers in cancers which can be analyzed and the treatment modality can be made over several days, clinical decisions have to be made within hours in septic patients, particularly, in elderly patients (1, 8). Hence, there is a demand in using clinical indicators to precision therapies instead of using complex biological characteristics. Finding out more associated clinical risk factors can help the early and easy identification of high mortality risk in septic patients.

Although compelling evidence from published literature has confirmed an increased risk of infection and sepsis in diabetic patients (9), whether diabetes would alter the mortality of septic patients is still conflicting (10–13). For instance, one study from Spain (12) suggested an optimal role of diabetes in decreasing the mortality rate of septic patients. However, several studies (10, 11, 14) highlighted that there was no association between the diabetic condition and the mortality of septic patients. Moreover, the relationship between admission blood glucose and clinical outcomes of sepsis also remains controversial (15–18). Thus, exploring the impact of admission blood abnormalities on the prognosis of septic patients coexisting with diabetes or not is of great clinical significance. It could not only provide more clinical-based evidence for making better therapeutic decisions, especially in terms of whether and when insulin should be applied, but also help clinicians investigate the underlying pathogenesis of sepsis.

In the current study, we accordingly aim to evaluate the impact of different admission blood glucose levels on 30-day mortality in septic patients with diabetes or not. Besides, we also aim to investigate the independent risk clinical factors in predicting the 30-day mortality of the septic population.

## Materials and Methods

### Data Source

The data of the present study were from a large-scale public database. All patients diagnosed with “sepsis,” “severe sepsis,” or “septic shock” in the Medical Information Mart for Intensive Care III (MIMIC III, version 1.4) database were potentially eligible for the present analysis. Specifically, the data regarding clinical characteristics of septic patients (icd9_code: 78552, 99591, and 99592) were obtained from the MIMIC III database, derived from a large, freely accessible critical care database comprising de-identified health records (58,976 hospitalization records) of ~50,000 patients who were admitted to the ICU of Beth Israel Deaconess Medical Center between June 2001 and October 2012 (https://mimic.physionet.org/) (19, 20).

### Ethics Approval

The use of the data derived from the MIMIC III database, provided by clinicians, data scientists, information technology personnel, and unidentified health information of patients, has not been deemed research of human subjects, and there was no requirement for individual patient consent because of the unidentified health information. Meanwhile, the protocol for this study was approved by Chongqing Medical University. Ethical approval was waived by the local Ethics Committee of the Chongqing Medical University in view of the retrospective nature of the study, and all the procedures being performed were part of the routine care.

### Patient Selection

**Figure 1:**
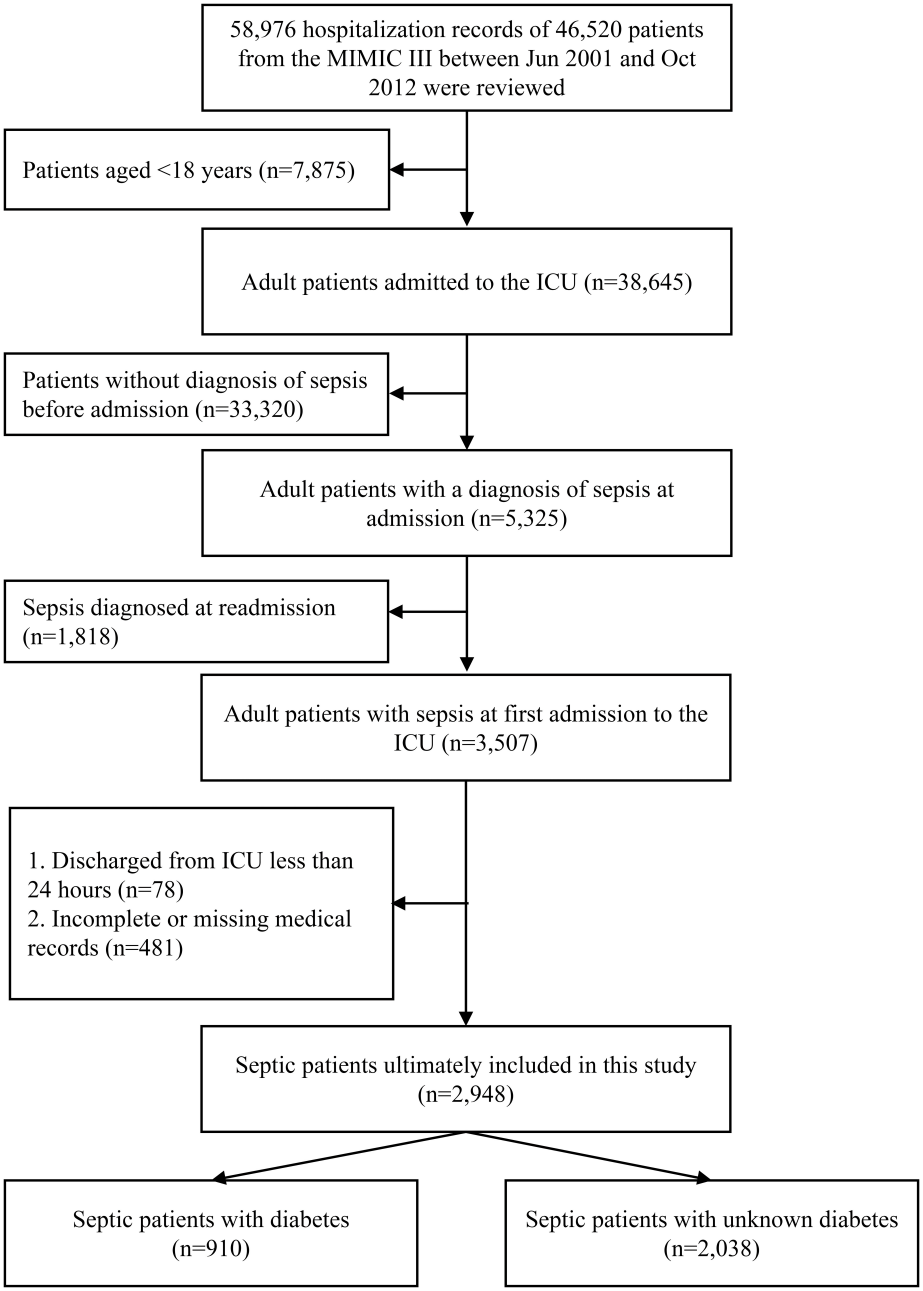
The patient selection process in the MIMIC III database. MIMIC III, the Medical Information Mart for Intensive Care III.

In the MIMIC III database, we retrospectively screened patients with a diagnosis of “sepsis” at admitting to the ICU. The specific patient-selection process is summarized in Figure 1.

### Diagnosis of Sepsis

The diagnosis of sepsis was according to The Third International Consensus Definitions for Sepsis and Septic Shock (sepsis-3) (21). Namely, the diagnosis of sepsis was based on the following criteria: (i) known or suspected infection; (ii) SOFA scores ≥2.

### Classification of Glucose Levels

The initial blood glucose levels were measured by using the blood gas analyzer when the patients were admitted to the ICU (22). We classified the glucose levels based on previously published studies, regardless of the presence or absence of diabetes (23, 24). The levels of blood glucose were divided into four subgroups: hypoglycemia: <70 mg/dl, normal: ≥70 and <140 mg/dl, hyperglycemia ≥140 and <180 mg/dl, and severity hyperglycemia ≥180 mg/dl.

### Definition of Diabetes

The diagnosis and classification of diabetes mellitus were following the international guidelines (25). As the data in the present study were derived from the MIMIC III program, the diagnosis of diabetes in septic patients was based on the codes “icd9_code:2535, 3572, 5881, 64800, 64801, 64802, 64803, 64804, 7751, V771, V1221, and V180.”

### Variable Evaluation

#### Clinical Baseline Information

The gender (female, male), age (>18 and ≤65 years, >65 years), race (white, black, and other), hypertension (yes and no), coronary heart disease (CHD, yes and no), chronic obstructive pulmonary disease (COPD, yes and no), diabetes (yes and no), chronic kidney disease (CKD, yes and no), heart rate, and blood oxygen saturation (Spo_2_) were reviewed.

#### Admission Severity Score

The SOFA score was analyzed individually according to the severity of system impairment (such as neurologic, renal, cardiovascular, respiratory, coagulation, and hepatic), each organ system got a score that ranges between 0 and 4. They were divided into three groups: total scores: ≥2 and ≤3, ≥4 and ≤5, and >5.

#### Admission Serological Indicators

The classifications of serological indicators were based on the references (low, normal, and high) displayed in the MIMIC III database. The white blood cell (WBC, normal: ≥4 and ≤10 k/ul), hemoglobin (Hb, normal: male > 120 g/L, female > 110 g/L), platelet (PLT, normal: ≥100 and ≤ 3,001 k/ul), potassium (K, normal: ≥3.5 and ≤5.5 mmol/L), base excess (BE, normal: ≥-3 and ≤3 mmol/L), anion gap (AG, normal: ≥8 and ≤16 mmol/L), lactic acid (Lac, normal: ≤2 mmol/L), blood urea nitrogen (BUN, normal: ≤21 mmol/L), serum creatinine (Scr, normal: male: <1.5 mg/dl, female: <1.0 mg/dl), albumin (ALB, normal: ≥3.5 g/dl), total bilirubin (Tbil, normal: ≤1.2 mg/dl), aspartate aminotransferase (AST, normal: ≤35 U/L), alanine aminotransferase (ALT, normal: ≤40 U/L), the percentage of neutrophils (NEU%, normal: 50–70%), and percentage of lymphocytes (LY%, normal; 20–40%) were screened out for constructing the database of the present study (Table 1).

**Table 1:** The demographic characteristics of septic patients with diabetes in different blood glucose levels.

**Variables**	**Subgroup**	**No. (%) of patients**				
		**<70 mg/dl** (*n* **= 39)**	**≥70 and <140 mg/dl** **(*n* = 332)**	**≥140 and <180 mg/dl** **(*n* = 204)**	**≥180 mg/dl** **(*n* = 335)**	***P***
Gender	Male	30 (76.9)	210 (63.3)	113 (55.4)	190 (56.7)	**0.025^a^**
	Female	9 (23.1)	122 (36.7)	91 (44.6)	145 (43.3)	
Age (years)	/	74 [62–79]^*^	69 [58–79]	71 [61–79]	68 [58–79]	0.466^c^
SOFA (score)	/	8 (6–13)	7 (4–10)	7 (4–10)	7 (4–9)	**0.019^c^**
Race	White	25 (64.1)	220 (66.3)	144 (70.6)	233 (69.5)	0.104^b^
	Black	4 (10.3)	51 (15.4)	18 (8.8)	28 (8.4)	
	Other	10 (25.6)	61 (18.4)	42 (20.6)	74 (22.1)	
CHD	No	32 (82.1)	266 (80.1)	157 (77.0)	259 (77.3)	0.712^a^
	Yes	7 (17.9)	66 (19.9)	47 (23.0)	76 (22.7)	
Hypertension	No	21 (53.8)	197 (59.3)	109 (53.4)	177 (52.8)	0.343^a^
	Yes	18 (46.2)	135 (40.7)	95 (46.6)	158 (47.2)	
COPD	No	39 (100.0)	326 (98.2)	202 (99.0)	329 (98.2)	0.911^b^
	Yes	0 (0)	6 (1.8)	2 (1.0)	6 (1.8)	
CKD	No	30 (76.9)	233 (70.2)	151 (74.0)	256 (76.4)	0.307^a^
	Yes	9 (23.1)	99 (29.8)	53 (26.0)	79 (23.6)	
WBC (k/uL)	/	14.1 [7.0–23.8]	12.3 [7.8–19.1]	14.3 [9.4–19.2]	14.6 [10.0–19.7]	**0.005^c^**
NEUT (%)	/	82.0 [72.0–88.0]	82.0 [76.0–87.0]	82.0 [78.0–88.0]	82.0 [76.0–88.0]	0.119^c^
LY (%)	/	6.0 [2.0–7.5]	7.5 [4.1–11.0]	7.5 [4.1–11.0]	7.5 [4.0–10.4]	0.233^c^
Hb (g/L)	/	9.60 [8.3–11.7]	9.7 [8.7–11.3]	10.0 [9.2–11.7]	10.7 [9.4–12.0]	** <0.001^c^**
PLT (k/uL)	/	180 [95–298]	179 [120–273]	194 [124–259]	216 [147–303]	**0.002^c^**
K (mmol/L)	/	4.0 [3.4–4.4]	4.0 [3.6–4.6]	4.2 [3.7–4.6]	4.2 [3.7–4.6]	0.223^c^
BE (mmol/L)	/	−6 [−10–4]	−3 [−5–0]	−3 [−5–0]	−3 [−7–0]	**0.008^c^**
AG (mmol/L)	/	16.0 [14.0–19.0]	15.0 [12.0–17.0]	15 [13.0–18.0]	16.0 [14.0–19.0]	**<0.001^c^**
Lac (mmol/L)	/	2.0 [1.1–3.8]	2.0 [1.3–2.7]	1.8 [1.2–2.7]	2.0 [1.5–3.2]	**0.004^c^**
BUN (mg/dl)	/	34.0 [21.0–46.0]	34.0 [20.0–52.0]	32.0 [19.0–52.0]	35.0 [23.0–52.0]	0.713^c^
Scr (mg/dl)	/	1.7 [1.2–2.7]	1.5 [0.9–2.8]	1.4 [1.0–2.6]	1.5 [1.1–2.4]	0.800^c^
ALB (g/dl)	/	2.5 [2.2–2.9]	2.6 [2.3–3.0]	2.6 [2.4–3.0]	2.7 [2.4–3.1]	0.104^c^
TBil (mg/dl)	/	1.2 [0.4–4.9]	1.0 [0.4–2.6]	0.8 [0.4–2.4]	0.7 [0.4–1.7]	**0.008^c^**
ALT (U/L)	/	31.0 [13.0–67.0]	33.0 [18.0–71.0]	32.0 [16.0–55.0]	33.0 [19.0–66.0]	0.255^c^
AST (U/L)	/	37.0 [23.0–126.0]	48.0 [27.0–98.0]	44.0 [23.0–82.0]	48.0 [25.0–105.0]	0.312^c^
Spo_2_ (mean)	/	97 [95–98]	97 [96–99]	97 [96–98]	97 [96–99]	0.208^c^
HR (mean)	/	90 [81–105]	85 [77–99]	90 [78–101]	91 [80–105]	**0.018^c^**
30-days mortality	No	20 (51.3)	220 (66.3)	134 (65.7)	241 (71.9)	**0.041^a^**
	Yes	19 (48.7)	112 (33.7)	70 (34.3)	94 (28.1)	

*CHD, coronary heart disease; COPD, chronic obstructive pulmonary disease; CKD, chronic kidney disease; SOFA, Sequential Organ Failure Assessment; WBC, white blood cell; Hb, hemoglobin; NEUT, neutrophil; LY, lymphocyte; PLT, platelet; K, potassium; BE, base excess, bicarbonate; AG, anion gap; Lac, lactic acid; BUN, blood urea nitrogen; Scr, serum creatinine (normal: male: <1.5 mg/dl, female: <1.0 mg/dl); ALB, albumin; Tbil, total bilirubin; ALT, aspartate aminotransferase; AST, alanine aminotransferase; HR, heart rate*.

* *Median [25th−75th percentile]*.

a *Pearson's Chi-squared test*.

b *Two-tail Fisher exact test*.

c *Kruskal–Wallis H-test*.

*Bold values indicate statistical significance (p < 0.05)*.

### Variable Selection

The following basic information, serological indicators, and the SOFA score scale from the MIMIC III database were screened out for investigating the risk factors associated with 30-day mortality in septic patients with diabetes or not: gender, age, race, SOFA, comorbidity, blood glucose, WBC, Hb, PLT, BE, AG, Lac, BUN, Scr, Tbil, ALT, and AST.

### Statistical Analysis

The primary endpoint of this study was 30-day mortality. The MIMIC software was applied to identify the patients who met the inclusion criteria in the MIMIC III program. Pearson-chi square test (minimal expected value > 5) and Fisher's exact chi-square test (minimal expected value ≤ 5) were conducted to compare the categorical variables. For continuity variables, data with a normal distribution (Mean± SD) were analyzed using Student's *t*-test and one-way ANOVA test, whereas data with non-normal distribution [Median (25th−75th percentile)] were analyzed using the Kruskal–Wallis *H* test. Univariate Cox regression analysis was used to identify the risk factors in septic patients. By using significant factors from univariate Cox regression analysis, the stepwise multivariate Cox regression analysis was further conducted to determine the independent prognostic factors in septic patients. Kaplan-Meier analysis was conducted to reveal the different 30-day survival probability in each subgroup, and the log-rank test was used to comparing the difference among these subgroups. A two-tailed *p*-value of <0.05 was defined as the criterion for variable deletion when performing backward stepwise selection. The univariate and multivariate Cox regression analyses were performed by using the SPSS for Windows (version 25; SPSS Inc., Chicago, IL, USA). The area under the receiver (AUC) operating characteristic (ROC) curve was calculated for evaluating the discrimination of the independent risk factors we determined.

## Results

### Demographic Characteristics of Septic Patients at Admitting to ICU

After excluding, a total of 2,948 septic patients were enrolled in this study within 30.9% (910/2,948) diagnosed diabetes (Supplementary Table 1). Compared with the non-diabetic group, the initial levels of blood glucose were much higher in the diabetic group (mean ± SD: 177.2±97.8 mg/dl vs. 131.0 ± 50.3 mg/dl, *p* < 0.001). Besides, a range of indicators was significantly different among diabetes and non-diabetic groups, such as the age distribution, race, comorbidity, peripheral blood indicators (WBC, NEUT%, LY%, Hb, PLT, K, BE, AG, BUN, Scr, ALB, and Tbil), and heart rate (*p* < 0.05). Nonetheless, among the diabetic group and non-diabetic groups, the 30-day mortality rate (34.2%) was equal (295/910 cases in diabetic patients and 661/2,038 cases in non-diabetic patients). In the diabetic group, patients with different admission blood glucose levels are presented with partially different clinical characteristics (Table 1). Partially in accordance with the diabetic group, similar clinical characteristics were also observed in the non-diabetic group (Table 2).

**Table 2:** The demographic characteristics of septic patients without diabetes in different blood glucose levels.

**Variables**	**Subgroup**	**No. (%) of patients**				
		**<70 mg/dl (*n* = 61)**	**≥70 and <140 mg/dl (*n* = 1,308)**	**≥140 and <180 mg/dl (*n* = 420)**	**≥180 mg/dl (*n* = 249)**	***P***
Gender	male	35 (57.4)	707 (54.1)	236 (56.2)	140 (56.2)	0.804^a^
	female	26 (42.6)	601 (45.9)	184 (43.8)	109 (43.8)	
Age (years)	/	60 [47–78]^*^	65 [52–79]	66 [54–80]	69 [57–79]	0.059^c^
SOFA (score)	/	9 (6–12)	6 (4–9)	7 (5–9)	8 (5–10)	**<0.001^c^**
Race	white	37 (60.7)	992 (75.9)	304 (72.4)	187 (75.1)	**0.038^a^**
	black	9 (14.7)	87 (6.6)	33 (7.8)	11 (4.4)	
	other	15 (24.6)	229 (17.5)	83 (19.8)	51 (20.5)	
CHD	No	57 (93.4)	1,152 (88.1)	369 (87.9)	221 (88.8)	0.626^a^
	Yes	4 (6.6)	156 (11.9)	51 (12.1)	28 (11.2)	
Hypertension	No	44 (72.1)	879 (67.2)	271 (64.5)	155 (62.2)	0.282^a^
	Yes	17 (27.9)	429 (32.8)	149 (35.5)	94 (37.8)	
COPD	No	61 (100.0)	1,281 (97.9)	412 (98.1)	239 (96.0)	0.197^b^
	Yes	0 (0)	27 (2.1)	8 (1.9)	10 (4.0)	
CKD	No	53 (86.9)	1,127 (86.2)	363 (86.4)	219 (88.0)	0.902^a^
	Yes	8 (13.1)	181 (13.8)	57 (13.6)	30 (12.0)	
WBC (k/uL)	/	11.3 [6.4–20.7]	12.0 [7.4–17.8]	12.7 [7.4–19.1]	14.2 [7.8–20.3]	**0.030^c^**
NEUT (%)	/	82.0 [74.0–85.0]	82.0 [73.0–87.0]	82.0 [72.0–88.0]	82.0 [75.0–89.0]	0.180^c^
LY (%)	/	7.5 [4.0–12.0]	7.5 [4.4–12.1]	7.5 [4.5–11.0]	7.5 [3.5–10.6]	**0.049**
Hb (g/L)	/	9.8 ± 2.1^**^	10.3 ± 1.9	10.7 ± 2.0	10.9 ± 2.2	**<0.001^d^**
PLT (k/uL)	/	143.0 [78.0–196.0]	176.0 [105.0–264.0]	178.0 [115.0–273.0]	194.0 [114.0–284.0]	**0.014^c^**
K (mmol/L)	/	4.2 [3.8–4.7]	3.9 [3.5–4.4]	4.0 [3.6–4.5]	4.0 [3.5–4.6]	**0.001^c^**
BE (mmol/L)	/	−7 [−12–2]	−3 [−6–0]	−3 [−7–0]	−3 [−7–1]	**<0.001^c^**
AG (mmol/L)	/	16.0 [14.0–20.0]	14.0 [12.0–17.0]	14.0 [12.0–17.0]	16.0 [13.0–19.0]	** <0.001^c^**
Lac (mmol/L)	/	2.4 [1.4–5.2]	2.0 [1.4–2.8]	2.1 [1.5–3.2]	2.4 [1.7–3.6]	** <0.001^c^**
BUN (mg/dl)	/	36.0 [21.0–45.0]	24.0 [15.0–41.0]	25.0 [16.0–42.0]	29.0 [19.0–51.0]	** <0.001^c^**
Scr (mg/dl)	/	1.7 [1.1–3.1]	1.1 [0.8–2.0]	1.2 [0.8–1.9]	1.3 [0.9–2.1]	** <0.001^c^**
ALB (g/dl)	/	2.5 ± 0.6	2.6 ± 0.5	2.6 ± 0.5	2.6 ± 0.6	0.471^d^
TBil (mg/dl)	/	2.1 [0.6–4.7]	1.0 [0.5–2.6]	0.9 [0.4–2.2]	0.8 [0.4–2.1]	**0.001^c^**
ALT (U/L)	/	58.0 [22.0–146.0]	33.0 [18.0–69.0]	33.0 [18.0–67.0]	35.0 [20.0–99.0]	**0.003^c^**
AST (U/L)	/	99.0 [41.0–271.0]	48.0 [26.0–99.0]	48.0 [27.0–100.0]	53.0 [29.0–111.0]	**0.001^c^**
Spo_2_ (mean)	/	97 [95–98]	97 [96–98]	97 [96–99]	97 [96–99]	0.073^c^
HR (mean)	/	102 [89–111]	93 [81–106]	94 [81–106]	94 [82–108]	**0.039^c^**
30-days mortality	No	26 (42.6)	917 (70.1)	276 (65.7)	158 (63.5)	**<0.001^a^**
	Yes	35 (57.4)	391 (29.9)	144 (34.3)	91 (36.5)	

*CHD, coronary heart disease; COPD, chronic obstructive pulmonary disease; CKD, chronic kidney disease; SOFA, Sequential Organ Failure Assessment; WBC, white blood cell; Hb, hemoglobin; NEUT, neutrophil; LY, lymphocyte; PLT, platelet; K, potassium; BE, base excess, bicarbonate; AG, anion gap; Lac, lactic acid; BUN, blood urea nitrogen; Scr, serum creatinine (normal: male: <1.5 mg/dl, female: <1.0 mg/dl); ALB, albumin; Tbil, total bilirubin; ALT, aspartate aminotransferase; AST, alanine aminotransferase; HR, heart rate*.

* *Median [25th−75th percentile]*.

** *Mean ± SD*.

a *Pearson's Chi-squared test*.

b *Two-tail Fisher exact test*.

c *Kruskal–Wallis H-test*.

d *One-way ANOVA test*.

*Bold values indicate statistical significance (p < 0.05)*.

### The Impact of Different Variables on 30-Day Mortality in Septic Patients Diabetes

**Figure 2:**
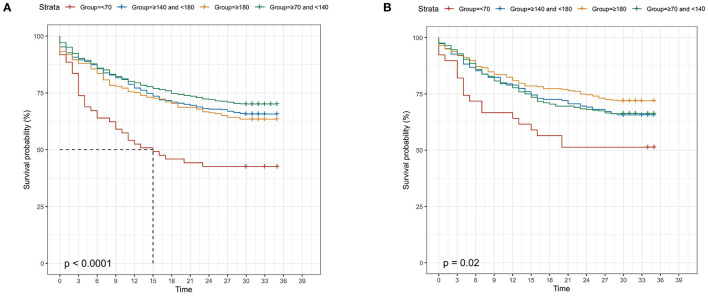
Kaplan-Meier curves for 30-day survival probability in septic patients, according to the admission blood glucose **(A)** in diabetic patients; **(B)** in non-diabetic patients.

In septic patients with diabetes, the Kaplan-Meier curves demonstrated that diabetic patients with septic with admission blood glucose <70 mg/dl (30-day survival probability: 51.3%, *p* = 0.020) had higher risk of 30-day mortality (Figure 2A). Additionally, severe SOFA score (30-day survival probability: 58.8%, *p* > 0.001), abnormal PLT (lower: 30-day survival probability: 54.4%, higher: 30-day survival probability: 64.6%; *p* > 0.001), higher Scr (30-day survival probability: 64.7%; *p* = 0.014), higher Lac (30-day survival probability: 64.2%, *p* > 0.001), higher BUN (30-day survival probability: 64.2%, *p* > 0.001), high AST (30-day survival probability: 64.4%, *p* = 0.006), elder age (30-day survival probability: 62.8%, *p* > 0.001), higher AG (30-day survival probability: 61.2%, *p* > 0.001), and higher Tbil (30-day survival probability: 61.3%, *p* > 0.001) also presented worse clinical prognosis outcomes (Supplementary Figure 1).

### Univariate and Multivariate Cox Regression Analyses

**Figure 3:**
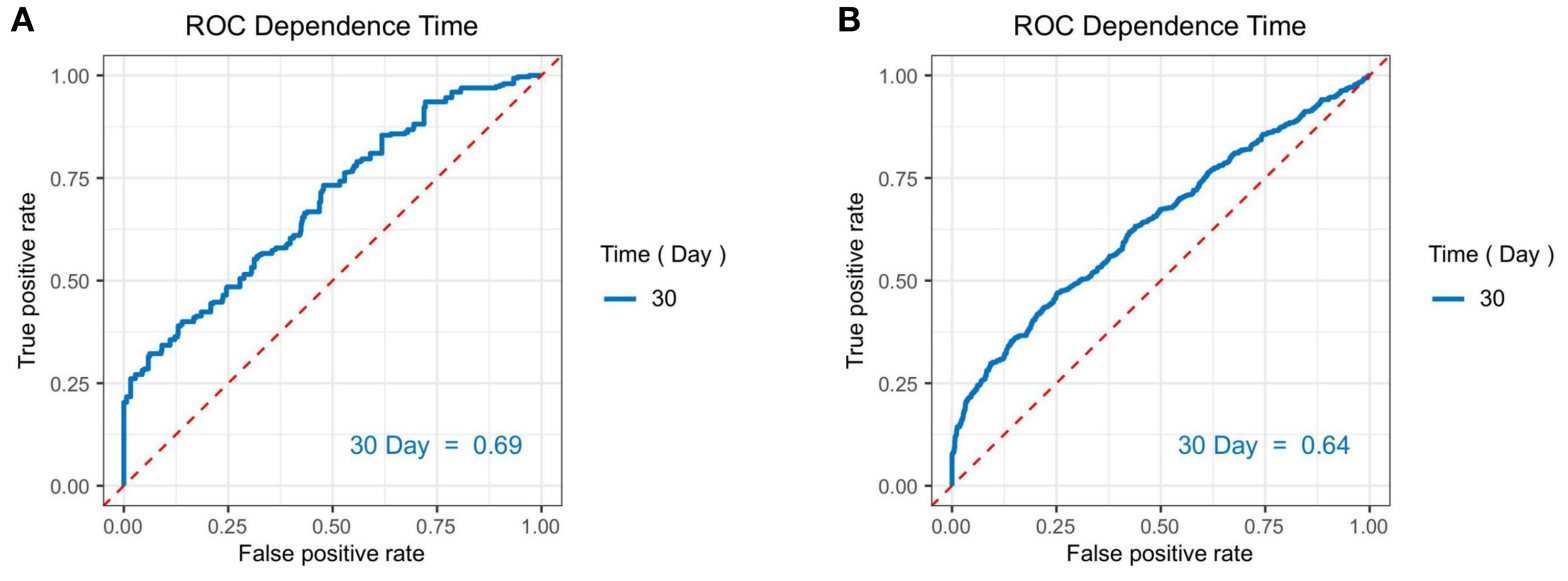
The area under the receiver (AUC) operating characteristic (ROC) curves of the independent risk factors we determined for predicting the 30-day mortality in septic patients **(A)** in diabetic patients; **(B)** in non-diabetic patients.

To explore the risk factors in predicting the 30-day mortality of septic patients with diabetes, the univariate Cox analysis was conducted. At univariate analysis, age (*p* < 0.001), SOFA score (*p* < 0.001), initial peripheral blood indicators, such as glucose (*p* = 0.024), AG (*p* < 0.001), Scr (*p* = 0.016), Lac (*p* = 0.001), BUN (*p* < 0.001), AST (*p* = 0.006), PLT (*p* < 0.001), and Tbil (*p* < 0.001) were significantly associated with the 30-day mortality. At multivariate analysis, age >65 years (hazard ratio (HR) = 1.53, 95% CI: 1.19–1.96; *p* = 0.001), SOFA score >5 (HR = 2.26, 95% CI: 1.45–3.52; *p* < 0.001), Lac >2 mmol/L (HR = 1.35, 95% CI: 1.04–1.76, *p* = 0.024), and PLT abnormality (<100 k/ul: HR = 1.49, 95% CI: 1.10–2.01; >300 k/ul: HR = 1.36, 95% CI: 1.02–1.80, *p* < 0.001) were the independent risk factors for predicting the worse 30-day survival probability in septic patients with diabetes (Table 3). Additionally, the AUC of the time-dependent ROC was 0.690 (Figure 3A).

**Table 3:** Univariate and multivariate Cox regression analyses of predictive variables correlated with 30-day mortality in septic patients with diabetes mellitus.

**Variables**	**Subgroup**	**Univariable**		**Multivariable**	
		**Hazard ratio**	***P***	**Hazard ratio**	***P***
Age (years)	≤ 65	Reference	**<0.001**	Reference	**0.001**
	>65	1.54 (1.20–1.96)		1.53 (1.19–1.96)	
Race	white	Reference	0.775		
	black	0.93 (0.63–1.36)			
	other	1.08 (0.81–1.43)			
Gender	female	Reference	0.861		
	male	0.97 (0.77–1.23)			
Comorbidity	0	Reference	0.471		
	1	0.94 (0.71–1.24)			
	≥2	1.12 (0.80–1.59)			
SOFA	≥2 and ≤ 3	Reference	** <0.001**	Reference	**<0.001**
	≥4 and ≤ 5	0.94 (0.55–1.60)		0.91 (0.53–1.56)	
	>5	2.82 (1.86–4.26)		2.26 (1.45–3.52)	
Glucose (mg/dl)	<70	1.69 (1.04–2.76)	**0.024**	1.40 (0.86–2.29)	
	≥70 and <140	Reference		Reference	0.144
	≥140 and <180	1.01 (0.75–1.36)		1.01 (0.75–1.37)	
	≥180	0.80 (0.61–1.06)		0.81 (0.61–1.08)	
(mmol/L)	< -3	0.99 (0.78–1.26)			
	≥-3 and ≤ 3	Reference	0.418		
	>3	0.72 (0.44–1.19)			
AG (mmol/L)	≤ 16	Reference	**<0.001**	Reference	0.249
	>16	1.52 (1.21–1.91)		1.16 (0.89–1.50)	
Scr (mg/dl)	normal	Reference	**0.016**	Reference	0.358
	high	1.34 (1.06–1.71)		0.87 (0.65–1.16)	
Lac (mmol/L)	≤ 2	Reference	**0.001**	Reference	**0.024**
	>2	1.55 (1.20–2.00)		1.35 (1.04–1.76)	
BUN (mg/dl)	≤ 21	Reference	** <0.001**	Reference	
	>21	1.69 (1.26–2.27)		1.31 (0.93–1.84)	0.122
ALT (U/L)	≤ 40	Reference	0.081		
	>40	1.22 (0.97–1.54)			
AST (U/L)	≤ 35	Reference	**0.006**	Reference	0.275
	>35	1.40 (1.10–1.78)		1.15 (0.89–1.50)	
Tbil (mg/dl)	≤ 1.2	Reference	**<0.001**	Reference	0.263
	>1.2	1.54 (1.22–1.94)		1.15 (0.89–1.49)	
WBC (k/uL)	<4	1.55 (0.98–2.46)			
	≥4 and ≤ 10	Reference	0.163		
	>10	1.09 (0.82–1.44)			
Hb (g/L)	normal	Reference	0.860		
	low	0.97 (0.76–1.26)			
PLT (k/uL)	<100	1.93 (1.45–2.58)		1.49 (1.10–2.01)	**0.008**
	≥100 and ≤ 300	Reference	** <0.001**	Reference	
	>300	1.36 (1.02–1.80)		1.40 (1.05–1.88)	

*SOFA, Sequential Organ Failure Assessment; WBC, white blood cell; Hb, hemoglobin (normal: male > 120 g/L, female > 110 g/L); PLT, platelet; BE, base excess, bicarbonate; AG, anion gap; Lac, lactic acid; BUN, blood urea nitrogen; Scr, serum creatinine (normal: male: <1.5 mg/dl, female: <1.0 mg/dl); ALB, albumin; Tbil, total bilirubin; ALT, aspartate aminotransferase; AST, alanine aminotransferase*.

*Bold values indicate statistical significance (p < 0.05)*.

### The Impact of Different Variables on 30-Day Mortality in Septic Patients Without Diabetes

In septic patients without diabetes, the Kaplan-Meier curves revealed that patients with admission blood glucose <70 mg/dl (30-day survival probability: 42.6%, *p* < 0.001) had the lowest survival rate, compared with other blood glucose levels (Figure 2B). Moreover, severe SOFA score (30-day survival probability: 59.7%, *p* < 0.001), higher Lac (30-day survival probability: 63.0%, *p* < 0.001), other race (30-day survival probability: 60.8%, *p* = 0.004), lower PLT (30-day survival probability: 57.0%, *p* < 0.001), higher Scr (30-day survival probability: 58.0%; *p* < 0.001), lower Hb (30-day survival probability: 65.6%, *p* = 0.004), higher Tbil (30-day survival probability: 61.2%, *p* < 0.001), higher BUN (30-day survival probability: 59.3%, *p* < 0.001), high AST (30-day survival probability: 63.5%, *p* < 0.001), high ALT (30-day survival probability: 64.3%, *p* = 0.002), elder age (30-day survival probability: 62.0%, *p* < 0.001), higher AG (30-day survival probability: 52.6%, *p* < 0.001), lower WBC (30-day survival probability:60.9%, *p* = 0.037) presented worse clinical prognosis outcomes (Supplementary Figure 2).

### Univariate and Multivariate Cox Regression Analyses

Regarding patients with the septic shock without diabetes, 14 variables, such as age (<0.001), race (*p* = 0.005), SOFA score (*p* < 0.001), initial peripheral blood indicators of glucose (*p* < 0.001), AG (*p* < 0.001), Scr (*p* < 0.001), Lac (*p* < 0.001), BUN (*p* < 0.001), ALT (*p* = 0.003), AST (*p* < 0.001), Tbil (*p* < 0.001), WBC (*p* = 0.040), Hb (*p* = 0.004), and PLT (*p* < 0.001) were remarkably associated with the 30-day mortality of septic patients without diabetes during the univariate analysis. At multivariate analysis, age >65 years (HR = 1.53, 95% CI: 1.29–1.81, *p* < 0.001), other race (HR = 1.30, 95% CI: 1.08–1.57, *p* = 0.004), SOFA score >5 (HR = 1.56, 95% CI: 1.19–2.04, *p* < 0.001), AG > 16 mmol/L (HR = 1.60, 95% CI: 1.35–1.89, *p* < 0.001), Lac > 2 mmol/L (HR = 1.61, 95% CI: 1.31–1.96, *p* < 0.001), BUN > 21 mmol/L (HR = 1.45, 95% CI: 1.17–1.79, *p* = 0.001), AST > 35 U/L(HR = 1.31, 95% CI: 1.07–1.60, *p* = 0.009), Tbil > 1.2 mg/dl (HR = 1.20, 95% CI: 1.01–1.43, *p* = 0.033), blood glucose <70 mg/dl (HR = 1.91, 95% CI:1.35–2.72, *p* = 0.003), and anemia (HR = 1.34, 95% CI: 1.12–1.61, *p* = 0.001) were the independent risk factors for predicting a worse 30-day survival probability in septic patients without diabetes (Table 4). The AUC of the time-dependent ROC was 0.640 (Figure 3B).

**Table 4:** Univariate and multivariate Cox regression analyses of predictive variables correlated with 30-day mortality in septic patients without diabetes.

**Variables**	**Subgroup**	**Univariable**		**Multivariable**	
		**Hazard ratio**	***P***	**Hazard ratio**	***P***
Age (years)	≤ 65	Reference	**<0.001**	Reference	**<0.001**
	>65	1.48 (1.27–1.73)		1.53 (1.29–1.81)	
Race	white	Reference	**0.005**	Reference	**0.004**
	black	0.82 (0.59–1.15)		0.78 (0.56–1.10)	
	other	1.31 (1.09–1.58)		1.30 (1.08–1.57)	
Gender	female	Reference	0.496		
	male	1.05 (0.90–1.23)			
Comorbidity	0	Reference	0.851		
	1	1.02 (0.86–1.19)			
	≥2	1.08 (0.82–1.41)			
SOFA	≥2 and ≤ 3	Reference	**<0.001**	Reference	**<0.001**
	≥4 and ≤ 5	1.17 (0.86–1.61)		0.97 (0.71–1.34)	
	>5	2.56 (2.00–3.28)		1.56 (1.19–2.04)	
Glucose (mg/dl)	<70	2.48 (1.75–3.50)	**<0.001**	1.91 (1.35–2.72)	**0.003**
	≥70 and<140	Reference		Reference	
	≥140 and<180	1.17 (0.96–1.42)		1.11 (0.92–1.35)	
	≥180	1.27 (1.01–1.60)		1.01 (0.80–1.28)	
BE (mmol/L)	< -3	0.94 (0.80–1.11)			
	≥-3 and ≤ 3	Reference	0.751		
	>3	0.91 (0.66–1.25)			
AG (mmol/L)	≤ 16	Reference	**<0.001**	Reference	**<0.001**
	>16	2.19 (1.88–2.55)		1.60 (1.35–1.89)	
Scr (mg/dl)	normal	Reference	**<0.001**	Reference	0.828
	high	1.92 (1.64–2.24)		1.02 (0.83–1.24)	
Lac (mmol/L)	≤ 2	Reference	**<0.001**	Reference	**<0.001**
	>2	1.96 (1.61–2.38)		1.61 (1.31–1.96)	
BUN (mg/dl)	≤ 21	Reference	**<0.001**	Reference	**0.001**
	>21	2.28 (1.91–2.71)		1.45 (1.17–1.79)	0.314
ALT (U/L)	≤ 40	Reference	**0.003**	Reference	
	>40	1.26 (1.08–1.47)		0.91 (0.76–1.09)	
AST (U/L)	≤ 35	Reference	**<0.001**	Reference	**0.009**
	>35	1.59 (1.36–1.85)		1.31 (1.07–1.60)	
Tbil (mg/dl)	≤ 1.2	Reference	**<0.001**	Reference	**0.033**
	>1.2	1.59 (1.34–1.88)		1.20 (1.01–1.43)	
WBC (k/uL)	<4	1.38 (1.07–1.79)		1.28 (0.98–1.67)	
	≥4 and ≤ 10	Reference	**0.040**	Reference	0.115
	>10	1.07 (0.90–1.29)		0.98 (0.82–1.19)	
Hb (g/L)	normal	Reference	**0.004**	Reference	**0.001**
	low	1.29 (1.08–1.53)		1.34 (1.12–1.61)	
PLT (k/uL)	<100	1.67 (1.40–1.99)		1.24 (1.01–1.51)	0.057
	≥100 and ≤ 300	Reference	**<0.001**	Reference	
	>300	1.07 (0.87–1.32)		1.18 (0.95–1.46)	

*SOFA, Sequential Organ Failure Assessment; WBC, white blood cell; Hb, hemoglobin (normal: male > 120 g/L, female > 110 g/L); PLT, platelet; BE, base excess, bicarbonate; AG, anion gap; Lac, lactic acid; BUN, blood urea nitrogen; Scr, serum creatinine (normal: male:<1.5 mg/dl, female:<1.0 mg/dl); ALB, albumin; Tbil, total bilirubin; ALT, aspartate aminotransferase; AST, alanine aminotransferase*.

*Bold values indicate statistical significance (p< 0.05)*.

## Discussion

Sepsis, as a major public health problem, is a life-threatening disease that is caused by a dysfunctional host response to infection and is associated with a high risk of death (2–4). In addition, sepsis and septic shock are associated with considerable long-term morbidity. Based on a remarkably large-scale population, Bauer et al. (6) demonstrated the average 30-day septic shock mortality was nearly 35%, and 90-day septic shock mortality was even higher and reached 38.5%. Overall, the average 30-day sepsis mortality was 24.4%, and 90-day sepsis mortality was 32.2%. The readmission rate of septic patients was 17.5% which is reported in recent works (1). While great progress has been made in critical care management, the clinical data from the US population (5) revealed neither the combined outcome of death nor discharge to hospice in septic patients changed significantly between 2009 and 2014. The main areas of controversy surrounding the early management of sepsis were the absence of definitive treatment strategies to change the course of the disease in these patients. Therefore, sepsis is still a threat that needs more comprehensive management on a global scale (3).

As one of the pivotal risk factors in developing infections, compelling evidence from published literature has confirmed the crucial but complex association between diabetes mellitus and sepsis (10, 12, 13, 26–29). Besides, diabetes could also increase the risk of 30-day readmission in patients with sepsis (1). Regarding the dysfunctional inflammatory response, the abnormalities of the host response, especially in red blood cell (RBC) deformability, neutrophil chemotaxis, adhesion, and intracellular killing defects that have been attributed to the effect of hyperglycemia were frequently observed in diabetic animals and humans (30–32). Although a range of works has investigated the influence of diabetes mellitus on prognosis in patients with sepsis, yet the findings were somewhat conflicting (10–12, 14). Specifically, whether the mortality of patients with sepsis was influenced by the presence of diabetes was an ongoing debate. In one study from two multicenter databases, Yende et al. (33) concluded that admission diabetes was associated with a higher risk of death following community-acquired pneumonia (HR = 1.3, after adjusting for the existing cardiovascular and renal disease). By conducting a large population-based prospective study, Donnelly et al. (28), however, determined that diabetes was significantly associated with increased risk of hospitalization for infection but not with higher 28-day mortality. Moreover, some works even showed that diabetic patients were significantly associated with lower in-hospital mortality (OR = 0.88) (12). Interestingly, Esper et al. (26) reviewed and analyzed the sepsis cases from the National Hospital Discharge Survey US, and they discovered that septic diabetic patients were less likely to develop acute respiratory failure (9 vs. 14%, *p* < 0.05) but more likely to develop acute renal failure (13 vs. 7%, *p* < 0.05). These remarkably different prognosis results reminded us of the complex association between diabetes mellitus and sepsis, particularly, in terms of the relation between different infection-associated sepsis and diabetes. For example, hyperglycemia-related metabolic acidosis might increase the movement of oxygen off of hemoglobin during the circulation and slightly relieve hypoxia (34), whereas the high bacterial burden and hyperglycemia would cause the disorders of renal tubular reabsorption and secretion (35, 36). However, our results presented that the diabetic condition did not affect the prognosis of sepsis but maintained a similar 30-day mortality rate in the two groups. Notably, with the deepening understanding of the pathogenesis of sepsis and diabetes, emerging studies paid attention to the correlation between blood glucose, sepsis, and sepsis shock (17). Therefore, we further, respectively, investigated the prognostic factors affecting the survival probability of septic patients with diabetes or not, especially in terms of admission blood glucose.

Reviewing the previous studies on exploring blood glucose concentrations and sepsis, we partially confirmed their results and take them a step further with the large population-based evidence. In the present study, we demonstrated the different risk patterns among septic patients with diabetes or not. After eliminating other enrolled confounders, hypoglycemia (blood glucose <70 mg/dl) was only significantly associated with increased 30-day mortality in septic patients without diabetes. On the other hand, neither diabetes nor non-diabetes septic patients had a higher mortality risk in severe hyperglycemia (>180 mg/dl) condition, compared with the euglycemia (≥140 and <180 mg/dl) group. It indicated that low levels of blood glucose potentially played a precursory role in reflecting the early dysfunction of self-regulation capability and inflammation-induced poor nutritional status, especially in patients without a history of diabetes mellitus. Notably, Furukawa et al. (37) showed both hypoglycemia and hypoglycemia combined with hypoalbuminemia were the independent risk factors in predicting the mortality of septic patients. However, their sample size (336 cases) was small and based on the single-center experience. While Kushimoto et al. (38) conducted a multicenter analysis and determined a similar result as ours, their sample size was relatively smaller than ours (1,158 cases vs. 2,948 cases) and they only focused on the severe septic patients which might not represent the overall septic patients. These limitations also existed in another recent study did by Ssekitoleko et al. in Uganda (39). The 30-day mortality rate in our study was 32.4% (956/2,948 cases) in the whole septic population without any difference among diabetic patients and non-diabetic patients, which was consistent with the mortality rate (31.4%) of Stegenga et al. (14) and relatively lower than the results (42.7%) derived from de Miguel-Yanes et al. (12). Alternatively, as reported in one prospective observational study (40), the level of blood glucose ≥ 200 mg/dl at admission was associated with higher 30-day mortality in sepsis (HR = 1.66), regardless of the concurrent with diabetes. Additionally, in another multicenter retrospective study, Stegenga et al. yielded the same conclusion that severe hyperglycemia itself instead of diabetes diagnosis could increase the in-hospital mortality of septic patients (14). Thus, the association between blood glucose levels and the prognosis of sepsis is still controversial and needs further exploration. Some objective factors including but not limited to different methods for measuring the blood glucose levels or the severity of the septic condition in the study population might contribute to the divergence of results in our study and others.

Nevertheless, it was a continuous management challenge for clinicians in tailoring the optimal glucose control goals for septic patients. In earlier findings (41), tight glucose controlling (TGC) ≤ 110 mg/dl would decrease morbidity and mortality among critically ill patients. Later on, Chin et al. (15), however, determined that elderly patients were more suitable to receive TGC strategy. Notably, in another later prospective study, the results of Waeschle et al. (42) did not support the beneficial role of TGC strategy in patients with severe sepsis and septic shock. Meanwhile, the TGC would instead increase the risk of hypoglycemia and hyperglycemia in these patients and further aggravate the septic episode. This conclusion was also yielded in one latest meta-analysis of randomized controlled trials (RCTs). Yamada et al. (43) determined a more robust conclusion that critically ill patients could not benefit from the TGC but had a significantly higher risk of hypoglycemia events.

Additionally, we also determined the independent risk clinical characteristics in promoting 30-day mortality among diabetic and non-diabetic patients. In diabetic subpopulation, elderly age (>65 years, HR = 1.53), higher SOFA scores (>5, HR = 2.26), higher Lac (>2 mmol/L, HR = 1.35), and PLT abnormality (<100 k/ul: HR = 1.49; >300 k/ul: HR = 1.40) were the independent risk factors in predicting the 30-day mortality. In the non-diabetic subpopulation, more underlying risk factors were identified, namely, other race (HR = 1.30), higher AG (>16 mmol/L, HR = 1.60), higher BUN (>21 mg/dl, HR = 1.45), higher AST (>35 U/L, HR = 1.31), higher Tbil (>1.2 mg/dl, HR = 1.20), and lower Hb (HR = 1.34 g/L) were the prognostics variables in predicting the 30-day mortality. AG was a pivotal indicator in evaluating the acid-base balance of critically ill patients. In our study, AG was not regarded as the risk factor in 30-day mortality in septic patients with diabetes but an opposite result was reached in patients without diabetes. It suggested that diabetes patients potentially had better compensation ability in dealing with metabolic acidosis. A recent observational study (44), however, revealed sodium bicarbonate infusion might not improve the outcome in overall septic patients with metabolic acidosis, but it was associated with improved survival in septic patients with late acute kidney disease (AKD) or severe acidosis. Whether septic patients without a history of chronic disease-related metabolic acidosis could benefit from the timely regulation of blood PH needs further exploration. Interestingly, we did not determine that multimorbidity was associated with a higher risk of mortality in diabetic and non-diabetic septic patients as Zador et al.'s report (45). The possible explanation was only four chronic diseases were enrolled for analysis, namely, CHD, COPD, hypertension, and CKD, which was less comprehensive than theirs. To date, there is still a long way to identify more comprehensively clinical factors affecting the pathophysiological changes and clinical outcomes of septic patients. Based on our results and recent works on investigating the association between clinical factors and sepsis, admission blood glucose played a much more important role in the prognosis of sepsis. Alternatively, it is still unclear whether this effect is actually due to the specific blood glucose concentration but confounding mixed variables that lead to hypoglycemia and subsequently worse outcomes. Nevertheless, the recent guidelines pointed out the promising target levels of blood glucose was range from 140 to 180 mg/dl (46).

In the present study, we provided new insights on exploring the association between admission blood glucose and the 30-day mortality in ICU septic patients, based on a large-scale population. Compared with other frequently used indicators like blood pressure, heart rate, and oxygen saturation (47–49), we, respectively, evaluated the role of admission blood glucose in the prognosis of diabetic and non-diabetic septic patients. Indeed, we determined admission blood glucose could be a novel complementary indicator for clinicians to predict the prognosis of septic patients, especially in non-diabetic patients.

In addition, we acknowledged there were some limitations in the present study. First, although this was a large-scale population-based study and the demographic characteristics were in line with previous studies of ICU septic patients, the retrospective nature of this study inevitably led to some selection bias. Second, while we adjusted for many potential confounders when explored the impact of blood glucose on 30-day mortality in septic patients, the possibility of residual confounders remained lacking in this study. Third, although we included as many variables as possible, other variables may have affected our results. For example, site of infection, isolation in cultures, treatment (fluid resuscitation, vasopressors, and inotropic), time at initiation treatment. Moreover, only the SOFA score was included for evaluating the condition of septic patients in the present study. Thus, more severity scores, like SAPS II or III or APACHE score, can be added. Also, some conventional indicators like C-reactive protein or procalcitonin, and the newly discovered immune biomarkers of sepsis (50) missing in the MIMIC III database could be further added to complement the existing works, which could help clinicians earlier identifying the high-risk mortality subpopulation. Lastly, we could not collect detailed information about all-cause death in septic patients with diabetes or not, as the present study was derived from the public database. Future robust evidence derived from large prospective randomized controlled studies is needed to validate our conclusions and obtain more detailed information on this topic.

## Conclusion

In summary, by using data from one large population-based public database, we determined admission blood glucose but not the diabetic condition was significantly associated with 30-day mortality in septic patients. Namely, admission blood glucose <70 mg/dl was an independent risk factor for predicting the mortality of septic patients without diabetes. We speculate admission blood glucose could be an important indicator for clinicians to make a more suitable and precise treatment modality in dealing with septic patients. Besides, we also discovered partially different individualized prognostic predictors in short-term mortality of septic patients with diabetes or not, especially in terms of peripheral blood indicators. These results need to be validated and strengthened in future works.

## Data Availability Statement

The original contributions presented in the study are included in the article/Supplementary Material, further inquiries can be directed to the corresponding author/s.

## Code Availability

The software application generated during and/or analyzed during the current study are available from the corresponding author on reasonable request

## Ethics Statement

Ethical approval was waived by the local Ethics Committee of the Chongqing Medical University in view of the retrospective nature of the study and all the procedures being performed were part of the routine care.

## Author Contributions

XW and LS contributed to the conception and design of the study. YM, XW, QW, HW, and SL organized the database. YM, XW, JY, HW, and SL performed the acquisition and statistical analysis. YM and XW were the major contributors in writing the first draft of the manuscript. All authors contributed to the article and approved the submitted version.

## Conflict of Interest

The authors declare that the research was conducted in the absence of any commercial or financial relationships that could be construed as a potential conflict of interest.

## Publisher's Note

All claims expressed in this article are solely those of the authors and do not necessarily represent those of their affiliated organizations, or those of the publisher, the editors and the reviewers. Any product that may be evaluated in this article, or claim that may be made by its manufacturer, is not guaranteed or endorsed by the publisher.

## Supplementary Material

Supplementary Figure 1 Kaplan-Meier curves for 30-day survival probability in septic patients, according to the admission indicators. **(A)** SOFA score; **(B)** platelet; **(C)** serum creatinine; **(D)** lactic acid; **(E)** blood urea nitrogen; **(F)** alanine aminotransferase; **(G)** age; **(H)** anion gap; **(I)** total bilirubin; **(J)** blood glucose. SOFA, the Sequential Organ Failure Assessment.

Supplementary Figure 2 Kaplan-Meier curves for 30-day survival probability in septic patients without known diabetes, according to the admission indicators. **(A)** blood glucose; **(B)** SOFA score; **(C)** lactic acid; **(D)** other race; **(E)** platelet; **(F)** serum creatinine; **(G)** hemoglobin; **(H)** total bilirubin; **(I)** blood urea nitrogen; **(J)** alanine aminotransferase; **(K)** aspartate aminotransferase; **(L)** age; **(M)** anion gap; **(N)** white blood cell. SOFA, the Sequential Organ Failure Assessment.

Supplementary Table 1 The demographic characteristics of sepsis patients in the present study.
