# Supplementary figures and images for: Admission Blood Glucose Is Associated With the 30-Days Mortality in Septic Patients: A Retrospective Cohort Study
Source: Front Med (Lausanne). 2021 Oct 28;8:757061. doi: 10.3389/fmed.2021.757061 (PMC8581133; doi:10.3389/fmed.2021.757061)

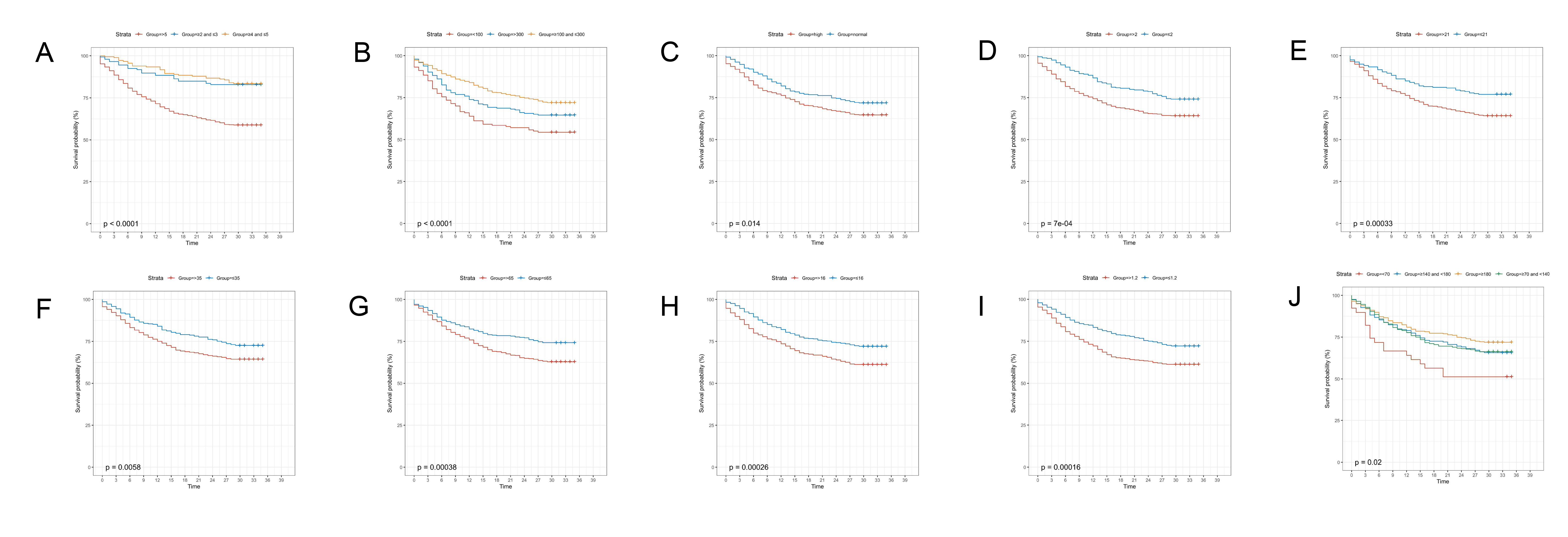

Supplement: Supplementary Figure 1 — Kaplan-Meier curves for 30-day survival probability in septic patients, according to the admission indicators. (A) SOFA score; (B) platelet; (C) serum creatinine; (D) lactic acid; (E) blood urea nitrogen; (F) alanine aminotransferase; (G) age; (H) anion gap; (I) total bilirubin; (J) blood glucose. SOFA, the Sequential Organ Failure Assessment. [file Image_1.TIF]

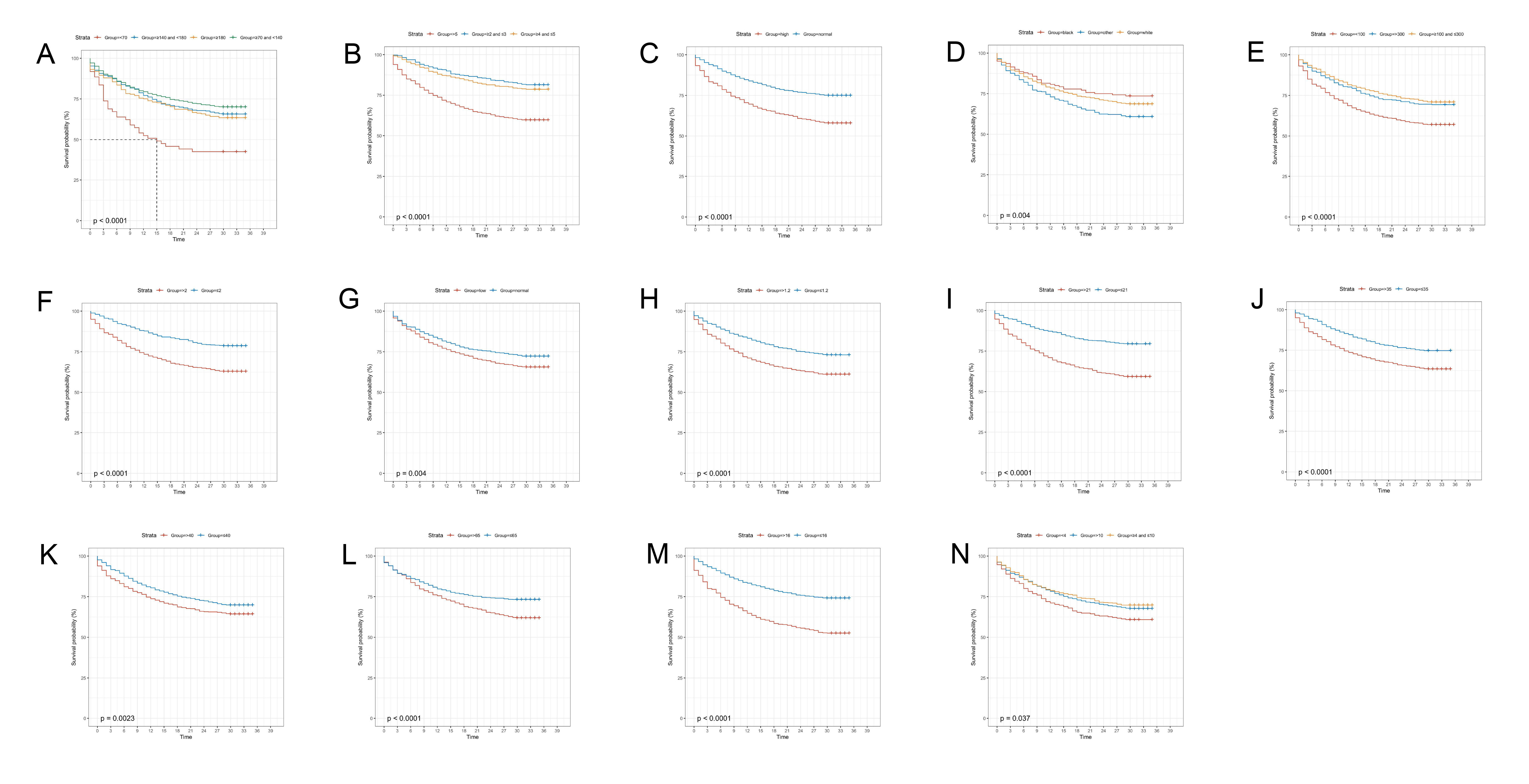

Supplement: Supplementary Figure 2 — Kaplan-Meier curves for 30-day survival probability in septic patients without known diabetes, according to the admission indicators. (A) blood glucose; (B) SOFA score; (C) lactic acid; (D) other race; (E) platelet; (F) serum creatinine; (G) hemoglobin; (H) total bilirubin; (I) blood urea nitrogen; (J) alanine aminotransferase; (K) aspartate aminotransferase; (L) age; (M) anion gap; (N) white blood cell. SOFA, the Sequential Organ Failure Assessment. [file Image_2.TIF]
