# Supplementary material for: Admission Blood Glucose Is Associated With the 30-Days Mortality in Septic Patients: A Retrospective Cohort Study
Source: Front Med (Lausanne). 2021 Oct 28;8:757061. doi: 10.3389/fmed.2021.757061 (PMC8581133; doi:10.3389/fmed.2021.757061)
Supplement: Supplementary Table 1 — The demographic characteristics of sepsis patients in the present study. [file Table_1.DOCX]

**Table S1.** The demographic characteristics of sepsis patients in the present study.

| **Variables** | **Subgroup** | **No. (%) of patients** | | | |
| --- | --- | --- | --- | --- | --- |
|  |  | **Total**  **(n=2,948)** | **Diabetes**  **(n=910)** | **Non-diabetes**  **(n=2,038)** | ***^#^P*** |
| **Gender** | male | 1,661(56.3) | 543(59.7) | 1,118(54.9) | ^a^**0.016** |
|  | female | 1,287(43.7) | 367(40.3) | 920(45.1) |  |
| **Age** (years) | / | ^*^65.74±16.48 | 68.25±13.88 | 64.61±17.41 | ^b^**<0.001** |
| **Race** | white | 2,142(72.3) | 622(68.3) | 1,520(74.6) | ^a^**<0.001** |
|  | black | 241(8.2) | 101(11.1) | 140(6.9) |  |
|  | other | 565(19.2) | 187(20.5) | 378(18.5) |  |
| **CHD** | No | 2,513(85.2) | 714(78.5) | 1,799(88.3) | ^a^**<0.001** |
|  | Yes | 435(14.8) | 196(21.5) | 239(11.7) |  |
| **Hypertension** | No | 1,853(62.9) | 504(55.4) | 1,349(66.2) | ^a^**<0.001** |
|  | Yes | 1,095(37.1) | 406(44.6) | 689(33.8) |  |
| **COPD** | No | 2,889(98.0) | 896(98.5) | 1,993(97.8) | ^a^0.257 |
|  | Yes | 59(2.0) | 14(1.5) | 45(2.2) |  |
| **CKD** | No | 2,432(82.5) | 670(73.6) | 1,762(86.5) | ^a^**<0.001** |
|  | Yes | 516(17.5) | 240(26.4) | 276(13.5) |  |
| **SOFA** (score) | ≥2 and ≤3 | 534(18.1) | 146(16.0) | 388(19.0) | ^a^0.146 |
|  | ≥4 and ≤5 | 579(19.7) | 181(19.9) | 398(19.6) |  |
|  | >5 | 1,835(62.2) | 583(64.1) | 1,252(61.4) |  |
| **WBC** (k/uL) | / | 14.39±10.23 | 15.20±10.51 | 14.03±10.08 | ^b^**<0.001** |
| **NEUT** (%) | / | 77.73±16.82 | 80.07±13.58 | 76.69±17.98 | ^b^**0.001** |
| **LY** (%) | / | 10.02±11.53 | 8.85±8.69 | 10.55±12.55 | ^b^**0.021** |
| **Hb** (g/L) | / | 10.45±2.03 | 10.41±2.04 | 10.46±2.03 | ^c^0.547 |
| **PLT** (k/uL) | / | 209.28±139.31 | 216.23±126.73 | 206.18±144.50 | ^b^**<0.001** |
| **K** (mmol/L) | / | 4.10±0.80 | 4.19±0.82 | 4.05±0.78 | ^b^**<0.001** |
| **BE** (mmol/L) | / | -3.81±5.54 | -3.76±5.67 | -3.84±5.49 | ^b^0.619 |
| **AG** (mmol/L) | / | 15.54±4.65 | 16.18±4.53 | 15.25±4.67 | ^b^**<0.001** |
| **Lac** (mmol/L) | / | 2.66±2.20 | 2.62±2.21 | 2.67±2.20 | ^b^0.147 |
| **BUN** (mg/dl) | / | 35.68±26.81 | 39.97±26.33 | 33.76±26.81 | ^b^**<0.001** |
| **Scr** (mg/dl) | / | 1.85±1.71 | 2.11±1.73 | 1.74±1.69 | ^b^**<0.001** |
| **ALB** (g/dl) | / | 2.65±0.56 | 2.70±0.56 | 2.62±0.55 | ^c^**<0.001** |
| **Tbil** (mg/dl) | / | 2.67±5.22 | 2.57±5.70 | 2.71±4.99 | ^b^**0.002** |
| **ALT** (U/L) | / | 129.89±455.06 | 123.31±406.14 | 132.82±475.35 | ^b^0.098 |
| **AST** (U/L) | / | 231.90±1,002.57 | 233.88±1,085.24 | 231.02±963.64 | ^b^0.058 |
| **Glucose** (mg/dl) |  | 145.34±71.81 | 177.27±97.81 | 131.08±50.32 | ^b^**<0.001** |
| **Spo2** (mean) | / | 96.60±3.46 | 96.82±2.91 | 96.50±3.68 | ^b^0.060 |
| **HR** (mean) | / | 92.94±17.51 | 90.34±16.62 | 94.10±17.77 | ^b^**<0.001** |
| **30-days mortality** | No | 1,992(67.6) | 615(67.6) | 1,377(67.6) | ^a^1.000 |
|  | Yes | 956(32.4) | 295(32.4) | 661(32.4) |  |

Abbreviation: CHD: coronary heart disease, COPD: chronic obstructive pulmonary disease, CKD: chronic kidney disease, SOFA: Sequential Organ Failure Assessment, WBC: white blood cell, Hb: hemoglobin, NEUT: neutrophil, LY: lymphocyte, PLT: platelet, K: potassium, BE: base excess, bicarbonate, AG: anion gap, Lac: lactic acid, BUN: blood urea nitrogen, Scr: serum creatinine (normal: male: <1.5mg/dl, female: <1.0mg/dl), ALB: albumin, Tbil: total bilirubin, ALT: aspartate aminotransferase, AST: alanine aminotransferase; HR: heartrate.

^*^Mean±SD, ***^#^***P: diabetes group compared with non-diabetes group.

^a^Pearson's Chi-squared test

^b^Kruskal-Wallis H-test

^c^Student t-test

Bold values indicate statistical significance (*p*<0.05)
